# Viral myocarditis in combination with genetic cardiomyopathy as a cause of sudden death. An autopsy series

**DOI:** 10.1186/s12872-024-03913-z

**Published:** 2024-05-29

**Authors:** Domitille Callon, Pierre Joanne, Laurent Andreoletti, Onnik Agbulut, Philippe Chevalier, Paul Fornès

**Affiliations:** 1 University of Reims Champagne Ardennes, INSERM, UMR-S1320 Cardiovir, Reims, France; 2 Biology Institute of Paris-Seine (IBPS), Biological Adaptation and Ageing, Sorbonne University, UMR CNRS 8256, INSERM U1164, Paris, France; 3 Forensic and Pathology Departments, Academic Hospital of Reims, Reims, France; 4 Virology Department, Academic Hospital of Reims, Reims, France; 5 Hospices Civils de Lyon, Lyon, France

**Keywords:** Myocarditis, Cardiomyopathy, Genetic, Sudden cardiac death, Molecular biology

## Abstract

Sudden cardiac death (SCD) is a major public health issue worldwide. In the young (< 40 years of age), genetic cardiomyopathies and viral myocarditis, sometimes in combination, are the most frequent, but underestimated, causes of SCD. Molecular autopsy is essential for prevention. Several studies have shown an association between genetic cardiomyopathies and viral myocarditis, which is probably underestimated due to insufficient post-mortem investigations. We report on four autopsy cases illustrating the pathogenesis of these combined pathologies. In two cases, a genetic hypertrophic cardiomyopathy was diagnosed in combination with Herpes Virus Type 6 (HHV6) and/or Parvovirus-B19 (PVB19) in the heart. In the third case, autopsy revealed a dilated cardiomyopathy and virological analyses revealed acute myocarditis caused by three viruses: PVB19, HHV6 and Epstein-Barr virus. Genetic analyses revealed a mutation in the gene coding for desmin. The fourth case illustrated a channelopathy and a PVB19/HHV6 coinfection. Our four cases illustrate the highly probable deleterious role of cardiotropic viruses in the occurrence of SCD in subjects with genetic cardiomyopathies. We discuss the pathogenetic link between viral myocarditis and genetic cardiomyopathy. Molecular autopsy is essential in prevention of these SCD, and a close collaboration between cardiologists, pathologists, microbiologists and geneticians is mandatory.

## Introduction

Sudden cardiac death (SCD) remains a major public health problem, worldwide, despite considerable advances in preventing strategies. Prevention is essential since the overall survival rate after cardiac arrest remains low < 5%, and more than 50% of sudden deaths occur in subjects with no cardiovascular history [1]. The overall estimate in the population is in the range of 300,000–350,000 SCDs per year in the USA [2, 3]. Event rates in Europe are similar to those in the USA [1]. In the young (< 35 years of age), the approximate incidence is 0.01 per 1000 per year [4].

Genetic cardiomyopathies, including structural cardiomyopathies and channelopathies, and viral myocarditis are the most frequent causes of SCD in this age group [1, 5, 6]. The reported prevalences of myocarditis are highly variable, ranging from 2 to 42% of cases [4]. Myocarditis is often underdiagnosed because diagnostic tools are not adequately performed [7]. It has been well established that histology, the so-called Dallas criteria, is an insufficient diagnostic method. In lymphocytic myocarditis, immunohistochemistry and molecular techniques such as polymerase chain reaction (PCR) are the gold standards for the diagnosis of viral myocarditis [4]. Human Enteroviruses (EV), Parvovirus B19 (PVB19) and Human Herpesvirus 6 (HHV6) are the most frequent viruses involved [8]. The viral load should be quantified, particularly for viruses such as PVB19, which is frequently an innocent bystander [7].

Cardiomyopathies are defined by the American Heart Association (AHA) as diseases of the heart muscle, mostly of genetic origin, causing heart failure and/or arrhythmia [9]. The likelihood that a SCD is caused by an underlying inherited disorder has led to the emerging role of genetic testing of DNA obtained at autopsy (also called “molecular autopsy”) [6, 7]. Thus, pathologists play an important role in the identification of families at risk, by reporting whether it is recommended to refer first-degree family members for clinical screening and/or to perform additional post-mortem genetic testing with cascade genetic screening, based upon the autopsy findings [6, 7].

Some studies have shown an association between genetic cardiomyopathies and viral myocarditis [10–15]. This association is probably underestimated due to the low proportion of autopsies performed in cases of SCD and inadequate investigation. Consequently, it is not known whether this association is coincidental or whether genetic cardiomyopathies predispose to opportunistic viral infections or reactivations. The question of viral-induced mutations is also raised.

In this context, we report on four autopsy-cases, illustrating the possible link between myocarditis and genetic cardiomyopathies.

## Materials and methods

In each case, a complete autopsy was performed. Hearts were grossly and histologically examined by a cardiovascular pathologist (PF), according to European guidelines [7]. Samples for histology (10 per case) were taken from the septum and left and right ventricular free walls.

Nucleic acids were extracted from frozen samples. Samples were first digested in proteinase K. Total nucleic acid (DNA and RNA) extractions were performed from tissue sample lysates using a NucliSens easyMAG device (BioMerieux). To detect Enteroviruses (EV) and all human Herpesviruses, PCR assays coupled with microarray hybridization analyses (Clart Entherpex V8.0, Genomica) were used. Quantitative PCR were used for EV [16] and HHV6 (R-Gene, Argene, Biomerieux) viral loads quantification. To detect human Parvovirus B19 (PVB19) infections, a specific real-time PCR assay (R-Gene, Argene Biomerieux) was used.

Next-Generation Sequencing (NGS) panels were performed according to French guidelines, based on the pathologist’s phenotype diagnosis. The following main genes were investigated: *MYH7, MYBPC3, TNNT2, TNNI3, MYL2* and *LMNA* genes. A larger panel includes 52 additional genes related to arythmogenic cardiomyopathy (ARVC), long QT syndrome, Amylosis, Brugada syndrome, Barth, Danon, Fabry, BAV, including *DES* and *RYR2* genes.

All experimental protocols were approved by a named institutional and/or licensing committee.

## Results

### Case #1

**Fig. 1:**
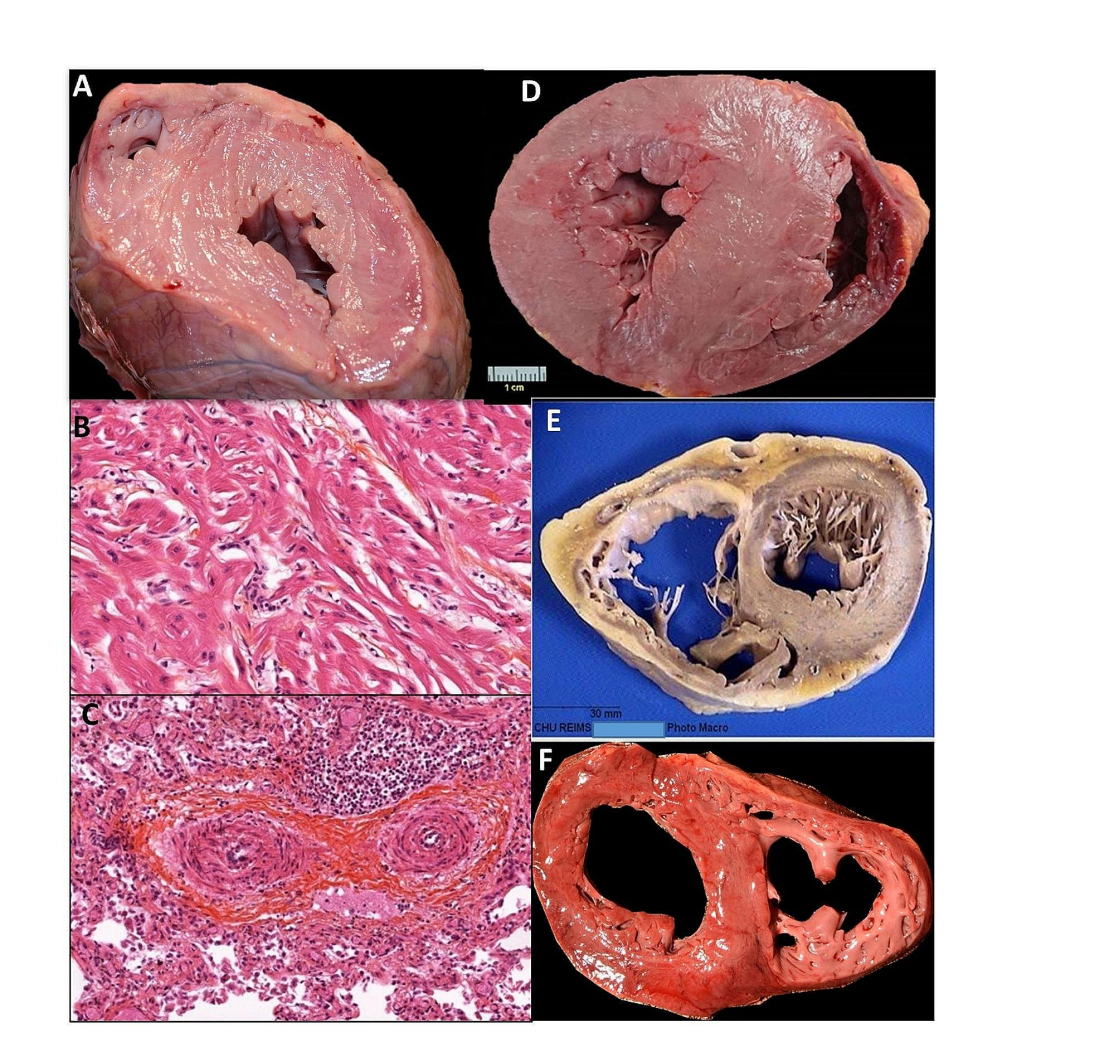
Autopsy and histology patterns. **A.** Gross pattern (case #1). **B.** Hypertrophic cardiomyocytes with disarray (case #1). **C**. Pulmonary arterial hypertension (case #1). Original magnification: x400 **D. E.** & **F.** Gross patterns (cases #2, 3 and 4)

A 4-year-old girl was found dead at her parents’ home. She had chickenpox with coughing and fever for 1 week, as her sister. Otherwise, she had no previous medical history. The autopsy revealed a typical hypertrophic cardiomyopathy (HCM) characterized by a huge heart weight (heart weight: 145 g; septal thickness 16 mm; free wall thickness 13 mm; body weight: 13,5 kg) with asymmetrical septal hypertrophy (Fig. 1A). Toxicology was negative.

Histologically, the cardiomyocytes were hypertrophic, with myocytes disarray (Fig. 1B). There was no inflammatory infiltrates. Immunohistochemical analysis with anti-CD3 (rabbit polyclonal, 1/200, Dako) and anti-CD68 (mouse monoclonal, clone KP1, 1/400, Dako) antibodies did not reveal T lymphocytes or macrophages, respectively. Histological examination of the lungs revealed severe lesions of pulmonary arterial hypertension (Fig. 1C). Other organs were histologically normal.

Virological analyses revealed Herpesvirus type 6 (HHV6) in both ventricles (171 and 206 copies/µg of nucleic acids extracted), in the spleen (253 copies/µg of nucleic acids extracted), in the liver (1552 copies/µg extracted nucleic acids) and Varicella-Zoster Virus (VZV) in the spleen.

The cause of the SCD was genetic HCM in combination with a HHV6-VZV co-infection. Severe pulmonary arterial hypertension complicated the HCM [17]. However, the parents did not give their consent to post-mortem genetic testing. Cardiological examinations were normal in first-degree relatives.

### Case #2

A 15-year-old boy died suddenly at home, at rest. He was treated with Propranolol for a known HCM, caused by a mutation of the cardiac myosin-binding protein C gene (*MYBPC3* gene; c3130C > T (p.Gln1044X). Variant class 5). The autopsy showed a typical asymmetrical hypertrophy (heart weight, 400 g; septal thickness 25 mm; free wall thickness 20 mm; 42 kg) **(**Fig. 1D). Toxicology was positive for Propranolol at therapeutic concentration.

Histological examination showed the characteristic patterns of HCM with myocytes disarray, fibrosis and intra-myocardial arterial dystrophy.

Virological analyses revealed Parvovirus B19 (PVB19) in both ventricles (67.4 copies/µg in left ventricle (LV) and 53.9 copies/µg in right ventricle (RV)) and HHV6 (10.6 copies/µg in LV). These viral loads are considered low.

The cause of SCD was a genetic HCM in combination with a PVB19/HHV6 co-infection. The father was also diagnosed with a HCM involving the same mutation.

### Case #3

A 60-year-old man, involved in a traffic car accident, was found dead in his car but was not injured. The autopsy revealed a dilated cardiomyopathy (DCM) (heart weight: 620 g; septal and free wall thicknesses, 18 mm) (Fig. 1E). Histologically, hypertrophic cardiomyocytes were associated with extensive interstitial and scarring fibrosis. There were inflammatory infiltrates consisting of T cells and macrophages infiltrates. Virological analyses revealed PVB19 (24.686 and 3000 IU/mL in LV and RV, respectively), Eptsein-Barr virus (EBV) and HHV6. These results indicated a severe active infection. Toxicology was negative.

Genetic analyses revealed a desminopathy caused by mutation of the gene coding for desmin -*DES* gene- located on chromosome 2 (c.1315G > A (p.Glu439Lys). Variant class 3). This false sense mutation has already been described in familial cases of cardiac desminopathy [18]. Mutations in the DES gene are involved in 1–2% of DCM are mainly found in the C-terminal domain. The myopathy was found in the mother, maternal grandmother and maternal uncle, plus SCD occured in a maternal aunt at the age of 50.

In summary, the patient had a genetic DCM in combination with a viral myocarditis caused by three viruses. The viral infection may have triggered the fatal arrhythmia. In the absence of genetic analysis, DCM would have been erroneously considered as a chronic form of viral myocarditis.

### Case #4

A 22-year-old man with no medical history was found dead at home. At autopsy, the heart and other organs were normal (heart weight: 410 g; septal and free wall thicknesses 15 mm. 1m81, 96 kg, BMI: 29.3) (Fig. 1F). Histology was normal. Immunohistochemistry was negative for CD3 and CD68. Virological analyses were positive for PVB19 and HHV6, with low viral loads, respectively 130 cp/µg and 1.5 cp/µg of extracted nucleic acids. Genetic testing on postmortem cardiac samples revealed a variant in the *RYR2* gene (false-sense mutation. Variant class 3) coding for Ryanodine receptor 2, with a missense mutation. This variant was considered responsible for Catecholaminergic Polymorphic Ventricular Tachycardia.

Toxicology revealed Tramadol at therapeutic concentration. Tramadol, due to its potential cardiotoxicity (long QT syndrome) might have been an additional trigger in combination with viral infection.

Family screening revealed that his brother died suddenly at 21 years old and that his 20-year-old sister had two unexplained faintnesses.

## Discussion

Our four autopsy cases illustrate the highly probable deleterious role of cardiotropic viruses in the mechanism of SCD in patients with genetic cardiomyopathies. In three cases (#1, 2 and 4), HHV6 and/or PVB19 were detected, with a low to moderate viral load without cellular inflammation. In case #3, viral myocarditis caused by three viruses with high viral loads was associated with genetic DCM.

Such associations have been reported in the literature, including PVB19 and channelopathies. Buob et al. published a case of sudden death in a 35-year-old man with typical EKG signs of Brugada syndrome in combination with PVB19 myocarditis [10]. In this case, the authors suggested that viral myocarditis was an arrhythmogenic substrate, or a trigger for fatal arrhythmia in Brugada syndrome. Juhasz et al. published a similar case of cardiac arrest in a 22-year-old man [13]. After resuscitation, he was diagnosed with Brugada syndrome. Two and a half months later, he died of PVB19 myocarditis. Salerno et al. reported on three patients with EKG abnormalities corresponding to Brugada syndrome, short QT syndrome and early repolarization syndrome in combination with viral myocarditis [14]. In both patients who survived, the abnormalities found on the EKG persisted, suggesting that they were not due to the myocarditis itself. These data suggested that channelopathies may predispose to ventricular arrhythmias in acute viral myocarditis. Frustaci et al. published a cohort of subjects with clinically unstable HCM with or without viral myocarditis diagnosed by histology, immunohistochemistry and PCR [11]. In this cohort, a high prevalence of positive viral detection was found, suggesting that HCM is a predisposing factor for myocardial viral infections. Bowels et al. published a series of 12 cases of arrhythmogenic right ventricular cardiomyopathy (ARVC) compared to 215 control cases [15]. The prevalence of positive viral detection was higher in cases of ARVC than in controls.

Clinical worsening phases of HCM and ARCV have been considered in relation with myocarditis [11, 15]. The variability in clinical expression of genetic cardiomyopathies within the same family are likely to be explained by myocarditis [19]. Our cases and other published case-reports raise the issue of a possible link between genetic cardiomyopathy/channelopathy and viral myocarditis. Further fundamental research is needed to investigate the mechanisms involved in this possible relationship, thereby exclude a random association [20].

## Conclusion

Viral myocarditis in combination with genetic cardiomyopathy is an underestimated cause of SCD in the young because of inadequate investigations. Our cases show that molecular biology is mandatory for both virology and genetics. A genetic substrate may be a predisposing factor for viral surimposed infections. The question of whether viral myocarditis might be involved in genetic mutation leading to genetic cardiomyopathies is also raised. Further fundamental reserach will be useful to address these issues.

## Data Availability

All data generated or analysed during this study are included in this published article.
